# Pilot prescription survey of antineoplastic agents: real‐world data from veterinary teaching hospitals in Japan

**DOI:** 10.1002/vms3.173

**Published:** 2019-05-17

**Authors:** Noriko Tanaka, Tsuyoshi Takizawa, Ryo Tanaka, Shozo Okano, Shinji Funayama, Toshio Iwasaki

**Affiliations:** ^1^ Department of Pharmaceutical Sciences Nihon Pharmaceutical University Saitama Japan; ^2^ Department of Pharmaceutical Sciences Chiba Institute of Science Choshi Chiba Japan; ^3^ Department of Veterinary Surgery Faculty of Veterinary Medicine Tokyo University of Agriculture & Technology Fuchu Tokyo Japan; ^4^ Department of Small Animal Surgery Veterinary Teaching Hospital School of Veterinary Medicine Kitasato University Towada Aomori Japan; ^5^ Department of Veterinary Internal Medicine Faculty of Veterinary Medicine Tokyo University of Agriculture & Technology Fuchu Tokyo Japan

**Keywords:** companion animals, electronic health records, prescription surveillance, antineoplastic agents, veterinary teaching hospital

## Abstract

The collection of real clinical records from veterinary practices and analysis of these records helps to establish evidence‐based veterinary medicine and further improves animal health and welfare. Prior to the collection of nationwide clinical records, we downloaded the data from the digital accounting systems of two veterinary teaching hospitals in Japan, and the prescriptions of antineoplastic agents were surveyed for a 5‐year period from 2009 to 2013. The ratio of the number of patients prescribed antineoplastic agents to the total number of prescriptions was <5% at both hospitals, and >80% of those patients were dogs. The overall number of prescriptions included more oral rather than injectable formations, whereas among antineoplastic agents, injectable formulations were prescribed more frequently at both hospitals. The most frequently prescribed agents were almost identical at both hospitals: platinum compounds, such as carboplatin and cisplatin (CDDP), vincristine and doxorubicin. The most frequently prescribed product combined with CDDP was doxorubicin at Hospital A. Antiemetic agents combined with CDDP included dexamethasone, ondansetron and metoclopramide, but these antiemetic agents were combined fewer than 10 times among 197 CDDP prescriptions. The prescription history, including the number of prescriptions, dosing intervals and combined medications, was provided by the survey. Although the present database consisted of data from two hospitals, our results indicate that a broad analysis can be conducted using integrated data from multiple hospitals and practices for further cohort studies.

## Introduction

Analysis of a database composed of electronic patient records enables veterinarians to establish evidence‐based veterinary medicine and select cost‐effective treatments, thus improving animal health and welfare. For example UK VetCompass and VetCompass Australia are nationwide big data collection platforms (O'Neill *et al*. 2014a,2014b; McGreevy *et al*. 2017). In Japan, both in veterinary practices and even in veterinary teaching hospitals, most patient records are still stored as paper‐based health records or separate data in individual medical devices without connection to hospital management systems (Tanaka 2011). However, according to a previous survey (Tanaka 2011; Miyamoto *et al*. 2013), account‐related items are electronically stored in hospital management systems in teaching hospitals. Using these data, we conducted a prescription survey of pharmaceuticals used in one teaching hospital and revealed that 76% of all prescriptions were drugs approved for humans (Tanaka *et al*. 2017). The antineoplastic agents (anticancer drugs) prescribed were all products approved for humans.

In companion animals, the incidence of age‐related disorders such as malignancies, cardiovascular diseases, obesity and diabetes increases as the animal ages (Inoue *et al*. 2015). The life expectancy of dogs has been extended in recent years in Japan(Suda 2011), which has resulted in an increased occurrence of age‐related disorders. Thus, medical products are needed that differ from those previously approved for veterinary use. Veterinary teaching hospitals in Japan are referral hospitals for primary care veterinarians. Various pharmaceuticals are used at teaching hospitals to treat older patient disorders, such as malignancies, thus promoting off‐label use of pharmaceuticals intended for human use. Several prescription surveys have been conducted at veterinary hospitals in many Western countries (Cave *et al*. 2007; Lobo *et al*. 2011; Wayne *et al*. 2011; Hughes *et al*. 2012; Carmo *et al*. 2017; McDougall *et al*. 2017). Almost all these surveys targeted prescriptions of anti‐infectious antibiotics and chemotherapeutics. Prescription surveys focusing on age‐related disorders treated by off‐label use of medicinal products for humans can provide real‐world treatment evidence such as the dose, dosing schedule, combined medical products and patient information.

In this study, the prescription data of two teaching hospitals that use the same digital system were surveyed to clarify the problems caused by merging the data. Prescription of antineoplastic agents was the focus of the present survey because the incidence of malignancies increases with age (Inoue *et al*. 2015b) and because among diagnostic categories, neoplasia represents the most frequent cause of death for dogs (Inoue *et al*. 2015). To establish more appropriate protocols, a survey of prescription histories using combination therapy is useful. Because many protocols for cancer treatments involve combinations of antineoplastic agents with different mechanisms(Cannon *et al*. 2015; Mauldin *et al*. 1988; Childress *et al*. 2018) and because antiemetics are used to reduce nausea and vomiting caused by antineoplastic agents (de la Puente‐Redondo *et al*. 2007; Kenward *et al*. 2017), the prescription history for each patient (especially for combination prescriptions) was also surveyed. This study is a pilot study to establish an appropriate survey method using digital health records to acquire real‐world veterinary evidence.

## Materials and methods

### Hospital selection criteria

Two of 16 Japanese veterinary teaching hospitals were selected based on the results of a questionnaire for hospital management systems (Tanaka 2011). These two referral hospitals for companion animals have the same digital accounting system, which is the most widely used hospital management system in Japan. The system is supported with a remote‐controlled system under a maintenance contract. Hospital A is located in the Tokyo suburbs, and Hospital B is located in the local provincial city suburbs.

### Prescription records

According to previously described methods (Tanaka *et al*. 2017), electronic prescription records were downloaded from Anicom Receptor (Anicom Pafe Inc., Tokyo, Japan), a veterinary hospital management system. Each hospital council approved the download of digital records from Anicom Receptor for use in the present surveillance study. The downloaded records included the following information: item number and treatment information, including the product name, prescription date, usage, dose, patient identification number (ID number), animal species, breed, sex, body weight, age, serial number and health record number. Personal data such as the patient owners' names, any information or ID numbers related to the owners, or the patients' names were not downloaded. The electronic file containing the downloaded data was sent to the author with a password. Data from 1 January 2009 to 31 December 2013 were analysed in this study.

### Product information

Among the downloaded treatment records, only medicinal products identified using the brand names were processed. The detailed method was described previously (Tanaka *et al*. 2017). Briefly, according to the Japan Standard Commodity Classification (Ministry of Internal Affairs and Communications, Statistic Bureau), the pharmaceutical therapeutic category (i.e. antineoplastic agents) was recognized in this study. Antineoplastic agents with another indication category were processed after an interview with the clinicians at the hospital regarding whether the product was used to treat cancer. In this study, products not indicated for use as antineoplastic agents were excluded even though veterinarians prescribed them to treat cancer patients (e.g. non‐steroidal anti‐inflammatory drugs [NSAIDs]).

### Data analysis

The prescription records and product name files were imported as data sets into SAS9.3 software (SAS Institute Japan Inc., Tokyo, Japan). The two data sets were match‐merged by treatment, thus attaching the brand name, generic name and classification code to the prescription records. Frequency distributions from the prescription data were examined. Infusion fluids were excluded from the present analysis because these products were not always recognized as independent accounting items by veterinary medical personnel in the hospitals (personal communication).

### Guidance

Because routinely collected health data were analysed in this study, RECORD guidance was used (Benchimol *et al*. 2015).

## Results

**Figure 1:**
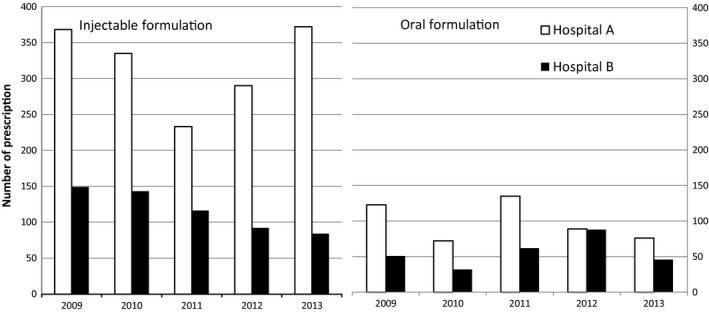
Annual changes in the numbers of antineoplastic agent prescriptions during the 5‐year study period. The open square indicates Hospital A, located in a Tokyo suburb; the closed square indicates Hospital B, located in the local provincial city suburbs. Each column on the left indicates the number of prescriptions for injectable formulations in a given year, and each column on the right indicates the number of prescriptions for oral formulations in a given year. No specific tendency was observed.

**Figure 2:**
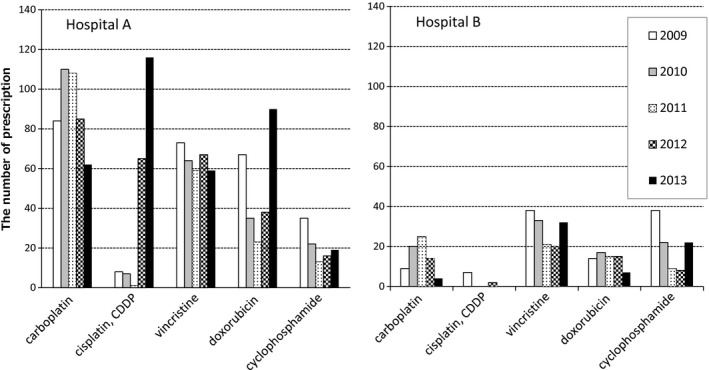
Annual changes in the number of prescriptions for frequently prescribed antineoplastic agents. The left figure shows the number of prescriptions at Hospital A, and the right figure shows the number of prescriptions at Hospital B. Each column indicates the number of prescriptions for each product in each year. Cyclophosphamide data include oral and injectable formulations. At Hospital A, the number of carboplatin prescriptions decreased from 2012, whereas the number of CDDP prescriptions increased.

The overall numbers of patient consultations were 10 681 and 8424 at Hospitals A and B, respectively, during the 5‐year study period (Table 1). The numbers of patients prescribed an antineoplastic agent were 377 (3.5% of all patients) and 194 (2.3% of all patients) at Hospitals A and B, respectively. More than 80% of all patients were dogs (5.3‐fold and 5.2‐fold more than cats at Hospitals A and B, respectively). More than 80% of the patients prescribed antineoplastic agents were also dogs (4.5‐fold more than cats at both hospitals). In total, 77 017 prescriptions were noted at Hospital A, including agents against pathogenic organisms, cardiovascular agents and other agents from 19 categories; 72.9% were oral products, although 76.0% of the antineoplastic agents prescribed were injectable (Table 2). In total, 43 806 prescriptions were noted at Hospital B, 55.2% of which were oral products, whereas 67.9% of antineoplastic agent prescriptions were injectable. All antineoplastic agents prescribed were products approved for human use. Any available veterinary antineoplastic agent was prescribed at either hospital during the study period, although toceranib is marketed in the United States and masitinib is in the EU. Figure 1 shows the annual changes in the number of prescribed antineoplastic agents at Hospitals A and B. No specific tendencies, such as an increase or decrease in prescription numbers, were observed. The number of prescriptions for each product is shown in Table 3. At Hospital A, the platinum compounds carboplatin and cisplatin (CDDP) were the most frequently prescribed agents, with 449 and 197 prescriptions, respectively (up to 30.7% of the total), followed by vincristine with 322 (15.3%) prescriptions and doxorubicin with 253 (12.0%) prescriptions. Both injectable and oral formulations of cyclophosphamide were used (105 and 305, respectively; up to 19.7%). At Hospital B, vincristine was the most frequently prescribed agent with 144 (16.6%) prescriptions, followed by cyclophosphamide with 99 injectable and 52 oral (up to 17.4%) prescriptions, oral chlorambucil with 90 (10.4%) prescriptions, platinum compounds including carboplatin with 72 prescriptions and CDDP with 9 prescriptions (together accounting for 9.3% of the total) and doxorubicin with 68 (7.8%) prescriptions. Nimustine was prescribed only at Hospital A, whereas procarbazine, methotrexate, bleomycin, etoposide, picibanil, lentinan and tretinoin were prescribed only at Hospital B. Because the number of patients prescribed antineoplastic agents was 1.8‐fold higher at Hospital A than that at Hospital B, the number of prescriptions for each product was higher at Hospital A than that at Hospital B with the exception of chlorambucil. CDDP was used much less frequently at Hospital B versus Hospital A. Among the products shown in Table 3, the annual change in the number of prescriptions for five products (carboplatin, CDDP, vincristine, doxorubicin and cyclophosphamide) is shown in Fig. 2. At Hospital A, the number of carboplatin prescriptions decreased from 2012, whereas the number of CDDP prescriptions markedly increased: a 14.5‐fold increase from 2009 to 2013. At both hospitals, carboplatin was prescribed for both dogs and cats, but CDDP was only prescribed for dogs. The prescription times and intervals of CDDP are shown in Table 4. The mean numbers of prescriptions were 5.0 and 2.3 at Hospitals A and B, respectively. Combined prescriptions were analysed for 32 CDDP‐prescribed dogs, and CDDP was prescribed a total of 197 times to the dogs at Hospital A. Among these 197 CDDP prescriptions, doxorubicin was additionally prescribed 50 times, and oral imatinib was prescribed two times. Injectable furosemide, a diuretic agent, was used in combination with CDDP two times. Antiemetics were used fewer than 10 of 197 times in combination with CDDP and comprised nine prescriptions for oral dexamethasone, two prescriptions for injectable ondansetron and one prescription for injectable metoclopramide. In addition to antiemetics, the gastrointestinal agent famotidine was prescribed eight times in combination with CDDP. The mean number of prescriptions for carboplatin in each individual patient was 5.0 ± 3.8, and the median number was 4.0 at Hospital A, the values of which were almost the same as those for CDDP prescriptions from 1 January 2009 to 31 December 2013.

**Table 1:** The number of consulted patients prescribed medicinal products

Hospital	Prescribed category	Total*	Dogs	Cats	Others†
A	All	10681 (100%)‡	8976 (84.0%)	1696 (15.9%)	9 (0.1%)
	Antineoplastic agents	377 (100%)	309 (82.0%)	68 (18.0%)	0 (0%)
B	All	8424 (100%)	7014 (83.2%)	1348 (16.0%)	62 (0.7%)
	Antineoplastic agents	194 (100%)	157 (80.9%)	35 (18.0%)	2 (1%)

* The number of patients was determined using the ID numbers.

† Horses from the college riding club and pigs used for veterinary practice at Hospital A; rabbits, guinea pigs, rodents and others at Hospital B.

‡ The ratio of the instances to the total number of prescriptions in each category is in parentheses.

**Table 2:** Numbers of oral and injectable product prescriptions

Hospital	Prescribed category	Number of prescriptions (%)*		
		Total	Injectable	Oral
A	All	77017 (100%)	20834 (27.1%)	56183 (72.9%)
	Antineoplastic agents	2103 (100%)	1598 (76.0%)	505 (24.0%)
B	All	43806 (100%)	19638 (44.8%)	24168 (55.2%)
	Antineoplastic agents	590 (100%)	590 (67.9%)	279 (32.1%)

* The ratio of the instances to the total number of prescriptions in each category is in parentheses.

**Table 3:** Numbers of prescriptions of antineoplastic agents

Oncology drug category and subcategory		Active ingredient	Hospital A		Hospital B	
			Injectable	Oral	Injectable	Oral
Alkylating agents	Mustard compounds	Cyclophosphamide	105	309	99	52
		Melphalan	na*	49	na	13
		Chlorambucil	na	49	na	90
	Nitrosoureas	Nimustine	58	na	0	na
		Lomustine	na	49	na	47
	Others	L‐asparaginase	66	na	63	na
		Procarbazine	na	0	na	7
Antimetabolite	Anti‐folics	Methotrexate	0	na	7	na
	Anti‐pyrimidines	Cytarabine	20	na	34	na
Antibiotics	Anthracyclines	Doxorubicin	253	na	68	na
		Mitoxantrone	2	na	13	na
	Others	Bleomycin	0	na	5	na
		actinomycin D	28	na	19	na
		mytomycin C	27	na	14	na
Anti‐microtubules	Vinca alkaloids	vincristine	322	na	144	na
		vinblastine	71	na	36	na
Platinum		cisplatin	197	na	9	na
		carboplatin	449	NA	72	na
Topoisomerase inhibitor		etoposide	na	0	na	8
Kinase inhibitor		imatinib	na	49	na	43
Nonspecific Immuno‐potentiator		picibanil	0	na	3	na
		lentinan	0	na	4	na
Others		tretinoin	0	na	19	na
Total			1598	506	590	279

* na, not available.

**Table 4:** Prescription analysis for cisplatin (CDDP)

Hospital	Total number of prescriptions	The number of CDDP‐prescribed animals		The prescription number per patient	
		Dogs	Cats	Mean ± SD*	Median (95% CI[Link])
A	197	32	0	5.0 ± 4.4	4 (2–5)
B	9	4	0	2.3 ± 0.4	2 (−)

* SD, standard deviation.

CI, confidence interval.

Among several antineoplastic kinase inhibitors, only imatinib was prescribed at both hospitals; it was prescribed 49 and 43 times at Hospitals A and B, respectively. The prescription histories for seven and five dogs receiving imatinib were examined at Hospitals A and B, respectively. A female miniature dachshund had the longest duration (44 hospital visits and 25 imatinib prescriptions at Hospital A). Twenty‐five ursodeoxycholic acid prescriptions, 24 famotidine prescriptions and 20 prednisolone prescriptions were combined with the 25 imatinib prescriptions. These three drugs (ursodeoxycholic acid, prednisolone and imatinib) were preferentially combined for other patients who were prescribed imatinib at Hospital A (data not shown). No prescription record for toceranib, a veterinary antineoplastic agent, was found at either hospital during our evaluation period.

## Discussion

Several surveillance studies have been conducted using electronic records, paper prescription records or Internet databases (Cave *et al*. 2007; Lobo *et al*. 2011; Radford *et al*. 2011; Wayne *et al*. 2011; Hughes *et al*. 2012; Kvaale *et al*. 2013; Carmo *et al*. 2017; McDougall *et al*. 2017). Most of these studies focused on the use of antimicrobial agents. Among them, Cave *et al*. (2007) examined the use of cytotoxic antineoplastic agents using a postal survey of UK veterinary practices. This study is the only report to analyse antineoplastic agents using medical treatment records. The ratios of patients with an antineoplastic agent prescription to the total number of patients were 3.5% and 2.3% at Hospitals A and B, respectively. However, the preferred formulation of antineoplastic agents was different from that of the overall patient group. This difference was observed because the overall group of patients mainly comprised patients prescribed agents against pathogenic organisms, cardiovascular agents, or agents affecting digestive organs as reported in a previous study (Tanaka *et al*. 2017), whereas antineoplastic agents were prescribed for <5% of the total population. Although oral products were preferably prescribed for the abovementioned categories (e.g. agents against pathogenic organisms), an injectable formulation was preferably prescribed for antineoplastic agents because the chemotherapeutic agents available for human patients with malignancy were injectable formulations before the development of oral kinase inhibitors.

In the study by Cave *et al*. (2007), the most widely prescribed antineoplastic agents in the UK practices were cyclophosphamide (65.4%) and vincristine (63.5%). Platinum‐based agents such as CDDP and carboplatin composed 8.3% of the prescriptions. In this study, cyclophosphamide and vincristine were also widely used products at both hospitals, but they were used less frequently compared with data in the UK: 19.7% and 17.4% for cyclophosphamide and 15.3% and 16.6% for vincristine at Hospitals A and B, respectively. However, platinum‐based agents were prescribed more frequently in this study (30.7% and 9.3% at Hospitals A and B, respectively) than in the UK according to a survey report in 2007 (8.3%). The median frequency of prescriptions ranged from once a month to once every 3 months in the UK (Cave *et al*. 2007). The median frequency of CDDP prescription was once every 4 weeks at Hospital A and once every 2 weeks at Hospital B. These discrepancies may be due to the country difference or differences between practices and referral hospitals. Big data analysis may allow evaluation of the reason for these differences. Combination protocols involving a platinum compound and doxorubicin have also been reported and are widely used to treat canine osteosarcoma; one such protocol is the CARBODOX6 protocol (Mauldin *et al*. 1988; Selmic *et al*. 2014). The use of combinations of antineoplastic agents with different mechanisms as well as antiemetics was also surveyed in this study. As shown in Fig. 2, carboplatin prescriptions decreased from 2012, whereas CDDP prescriptions increased at Hospital A. Carboplatin was switched to CDDP because of the prices of the two products, with CDDP as the less expensive agent (personal communication). Doxorubicin was the product combined most frequently with CDDP. Treatment‐related adverse events associated with CDDP were somewhat different from those associated with carboplatin, such as the contraindication in cats because of dose‐related severe pulmonary toxicities, renal impairment or myelosuppression (Plumb 2015). Carboplatin is less nephrotoxic and less emetic than CDDP in animals as well as in humans (Rose & Schurig 1985; Multinational Association of Supportive Care in Cancer, 2016). In addition to the general adverse events associated with chemotherapeutics, such as myelosuppression, CDDP dosing requires hydration to inhibit renal impairment progression (Plumb 2015), and the combined use of several antiemetics to control emesis is also recommended in veterinary medicine (de la Puente‐Redondo *et al*. 2007; Rau *et al*. 2010; Kenward *et al*. 2017). A worldwide online survey by the American Society of Clinical Oncology showed that the development of antiemetics was voted as one of the ‘Top 5 Advances in 50 Years of Modern Oncology' (American Society of Clinical Oncology, 2014). The use of antiemetics improves patients' quality of life and allows them to tolerate an adequate dose intensity of antineoplastic agents (Multinational Association of Supportive Care in Cancer, 2016). In this study, antiemetics were infrequently combined with 197 CDDP prescriptions, with only two ondansetron injection prescriptions (1%) and one metoclopramide injection prescription (0.5%). Oral dexamethasone was prescribed in combination with CDDP nine times (4.6%), but the prescription purpose of dexamethasone could not be determined (personal communication). Other than antiemetics, famotidine was used eight times.

A prescription history survey can be useful to establish a standard therapy protocol, indicating the importance of a combination survey. The present survey shows that there was no prescription data for the antineoplastic product approved for veterinary use, toceranib. The supply of toceranib to the hospitals was restricted during the study period; since then, however, the product has become available for prescription at these hospitals (personal communication). Analysis of the use of antineoplastic products approved for veterinary use will become possible in the near future. Imatinib was the only kinase inhibitor prescribed at both hospitals. KIT tyrosine kinase is the target protein for imatinib as well as toceranib, an antineoplastic product approved for veterinary use (Heinrich *et al*. 2000; Pryer *et al*. 2003; London *et al*. 2012). The prescription history of the dog with the longest term consisted of 44 hospital visits and 25 imatinib prescriptions at Hospital A. Because successive treatments are essential for kinase inhibitors, this long treatment experience with imatinib revealed that the patient tolerated the drug well. The data can be referenced for the future use of newly marketed antineoplastic products approved for veterinary use, such as toceranib and masitinib, which are similar to imatinib. In the present survey, a prescription history survey was conducted for each individual patient. A prescription history survey combined with additional haematological or biomarker examination data can provide real‐world treatment evidence and facilitate the development of standard protocols in veterinary medicine.

The present database included data from two hospitals, and the same analytical methods were applied for merged data from the two hospitals without problems, indicating that a broad analysis can be conducted using integrated data from multiple hospitals and practices. VetCompass Australia, a nationwide big data collection system for veterinary science, was started in 2017(McGreevy *et al*. 2017); likewise, VetCompass has been running in the UK for 7 years (O'Neill *et al*. 2014a,2014b). Such a nationwide data collection system based on real veterinary health records can promote cohort studies and health surveillance studies.

## Source of funding

This study was partially funded by Meiji Seika Pharma Co. Ltd., Tokyo.

## Conflict of interest

The authors declare no conflict of interest.

## Ethical statement

The authors confirm that the ethical policies of the journal, as noted on the journal's author guidelines page.

## Contributions

All authors agree to their contribution as shown below. The corresponding author, N. Tanaka, keeps their statements. All authors have read and approved the final manuscript.

N. Tanaka: Planned and supervised the research project, performed the research and wrote the paper.

T. Takizawa: Performed the statistical analysis and handled the SAS software.

R. Tanaka: Arranged to download data from the hospital system and reviewed the results and the paper.

S. Okano: Arranged to download data from the hospital system and reviewed the results and the paper.

S. Funayama: Supported the research progress, arranged necessary facilities and reviewed the paper.

T. Iwasaki: Arranged to download data from the hospital system and reviewed the results and the paper.
